# Lugol’s Iodine-Enhanced Micro-CT: A Potential 3-D Imaging Method for Detecting Tongue Squamous Cell Carcinoma Specimens in Surgery

**DOI:** 10.3389/fonc.2020.550171

**Published:** 2020-10-21

**Authors:** Cheng-Wan Xia, Rong-lin Gan, Jiong-ru Pan, Shi-qi Hu, Qun-zhi Zhou, Shen Chen, Lei Zhang, Qin-Gang Hu, Yu-Xin Wang

**Affiliations:** ^1^Department of Oral and Maxillofacial Surgery, Nanjing Stomatological Hospital, Medical School of Nanjing University, Nanjing, China; ^2^Department of Stomatology, The Suzhou Hospital That Is Affiliated to the Nanjing Medical University, Suzhou, China; ^3^Department of Oral Pathology, Nanjing Stomatological Hospital, Medical School of Nanjing University, Nanjing, China

**Keywords:** I_2_-IK, micro-CT, pathological examination, surgical margin, TSCC specimens

## Abstract

**Background:**

A positive surgical margin (PSM) following oral cancer resection results in local recurrence and poor prognosis. Mono-block tumor specimens, especially from the tumor base, are difficult to evaluate. This inaccurate sampling ultimately leads to a false pathological diagnosis. Lugol’s iodine (I_2_-IK)-enhanced micro-CT is an emerging method to image tumor specimens. This study explores the feasibility of I_2_-IK-enhanced micro-CT to evaluate the surgical margin for tongue squamous cell carcinoma (TSCC) specimens and to further seek optimal staining parameters.

**Methods:**

Rabbit tongue tissues and human TSCC samples were imaged *via* I_2_-IK-enhanced micro-CT. The optimal I_2_-IK concentration and staining time were determined before clinical application using tissue shrinkage, micro-CT image quality, and effect on pathological diagnosis as assessment criteria. Next, 6 TSCC specimens were used to verify the process feasibility of surgical margin imaging with the optimal parameters. Finally, the possible reason by which I_2_-IK could enhance micro-CT imaging was validated *in vitro*.

**Results:**

I_2_-IK staining influenced specimen shrinkage, micro-CT image quality, and pathological image quality in a concentration- and time-dependent manner. After comprehensively considering these indicators, 3% I_2_-IK staining for 48 and 12 h were found to be optimal for rabbit tongue tissues and TSCC samples, respectively. This method could provide a detailed 3-D structure of TSCC samples compared with H&E sections. Moreover, tumor and normal tissues could be differentiated by their glycogen content, which has high affinity with I_2_-IK.

**Conclusions:**

I_2_-IK-enhanced micro-CT could, thus, indicate the tumor margin and assist pathological sampling in patients with TSCC postoperation.

## Introduction

Oral squamous cell carcinoma (OSCC) is one of the most common cancers worldwide, especially in developing countries (1). Surgical tumor resection remains as the first treatment for patients with OSCC, but the 5-year survival rates continue to be approximately 60% despite the combination of surgical treatment and adjuvant therapies in recent decades (2). Local recurrence is the main reason for treatment failure (3). Among OSCC cases, tongue squamous cell carcinomas (TSCC) has the highest local recurrence rate (29.6%) due to its high-grade malignancy and strong infiltration into the lingual muscle (4).

The principle of en-bloc resection has been standard in surgical oncology. Surgical margins on mono-block specimens after tumor resection need to be evaluated for solid organ cancers. In fact, the positive surgical margin (PSM) rate for oral cavity cancer has been reported to be 12.75%—the highest overall PSM rate among all-site tumors (5). Furthermore, the survival rate of patients with PSM is significantly lower than that of patients with a negative surgical margin (6). Therefore, accurate evaluation of surgical margin is important to improve the prognosis of patients with TSCC. At present, clinically and/or pathologically satisfactory margins are determined intraoperatively and postoperatively by surgeons or pathologists. However, the samples obtained from the glossectomy specimen or the tumor bed for surgical margin evaluation are subjective and/or arbitrary; therefore, the local recurrence rate remains 10%–30% even when the surgical margin is confirmed as satisfactory by pathology (7, 8). In other words, uncertain surgical margins are closely associated with difficulty in the examination of mono-block specimens.

Serial whole-mount sectioning is an effective way to examine mono-block specimens; it is also helpful to decide reasonable treatment options based on the NCCN guidelines. However, this is impractical for patients with TSCC and for clinicians because of its high cost and time consumption (9). Near-infrared fluorescent imaging using indocyanine green (ICG) can detect the surgical margin during surgery and has been applied in breast, colon, lung, and several cancer types to position the PSM successfully (10–14). However, for patients with OSCC, secondary inflammation caused by bacteria inhabiting the oral cavity may produce a false PSM due to enhanced permeability and retention effects (15). Moreover, the 1–3 mm penetration depth of ICG fluorescence is limited to the evaluated surgical margin on specimens (14).

With the development of medical imaging, spatial resolution of micro-CT has reached the micron level. Thanks to the significant density difference between the tumor and background tissues, micro-CT is feasible to locate the tumor and estimate the surgical margin on mono-block specimens of breast cancer (16). For TSCC specimens, micro-CT imaging has not been used to evaluate the surgical margin because the density of tumor tissues and the surrounding tissues is similar. Lugol’s iodine (I_2_-IK)-enhanced micro-CT is an emerging method to solve this dilemma as it can show the fine anatomic structure of various tissue samples in various animals (17). Furthermore, in surgical oncology, Apps et al. demonstrate that I_2_-IK-enhanced micro-CT imaging provides detailed 3-D structural information of adamantinomatous craniopharyngioma in volumes with isotropic voxel sizes of 4–6 microns, and these results match well with H&E and immunohistochemical (IHC) sections (18). Thus, owing to nondestructive rapid imaging (19, 20) and high resolution, I_2_-IK-enhanced micro-CT has become a promising method to detect surgical margins during operation.

In this study, I_2_-IK-enhanced micro-CT was used for the first time to examine TSCC samples. First, the parameters, including I_2_-IK concentration and staining time, were explored in rabbit tongue tissues and in human TSCC samples using tissue shrinkage, quality of micro-CT image, and subsequent effect on pathological examination as the evaluation indicators. Next, 6 human TSCC specimens were used to verify the feasibility of this process for surgical margin imaging based on the optimal parameters. Finally, cytological experiments were conducted to explain the possible reasons underlying I_2_-IK-enhanced micro-CT imaging.

## Materials and Methods

### Rabbit Tongue Tissues and Human TSCC Samples

Fifteen New Zealand white rabbits (male, 2.5 kg) were purchased from the Comparative Medical Center of Yang Zhou University. After the rabbits were euthanized, a total of 27 tissues from the front of the tongue were cut off; 15 of these were used for I_2_-IK-enhanced micro-CT imaging, and the remaining 12 tissue blocks were used to verify the consistency of volume measurement between the drainage method and the micro-CT 3-D reconstruction method.

For human TSCC sample acquisition, this study was first approved by the medical ethics committee of the Institute Affiliated Stomatology Hospital, the Nanjing University Medical School. Then, 21 samples were acquired from 3 patients with TSCC. The samples were acquired from the junction between the tumor and normal tissues. All 3 patients provided a signed informed consent form. Fifteen samples from the same patient were used to determine the optimal parameters for I_2_-IK-enhanced micro-CT imaging. The remaining 6 samples from 2 patients were used to further verify the feasibility of I_2_-IK enhanced micro-CT to evaluate the status of the surgical margin in TSCC.

### I_2_-IK Staining

I_2_-IK (W/V, the concentration of iodine was 15%) was purchased from Shandong lvying Chemical Technology Co. Ltd., China. Various concentrations including 1%, 3%, 5%, and 7% (w/v, iodine concentration) of I_2_-IK were prepared by diluting the I_2_-IK mother liquor with 4% formalin solution. All I_2_-IK solutions were preserved at room temperature away from light. After fixation with formalin for 12 h, both rabbit tongue tissues and human TSCC samples were stained with different concentrations of I_2_-IK for 72 and 48 h, respectively, in 10-ml brown bottles. During the staining period, these samples were scanned by micro-CT every 12 h.

### Micro-CT Imaging and Quality Assessment of CT Image

A small animal micro-CT (Hiscan XM, Suzhou Heisfeld Information Technology Co. Ltd., China) was used for all CT imaging. The micro-CT scan parameters were as follows: power: 8 W, voltage: 60 V, electric current: 133.3 μA, detector mode: binging 2*2, slice thickness: 50 μm, and repetition time: 75 ms. All CT image data were processed using the SeProcessPro Version.1 software (Version 1.0, Suzhou Heisfeld Information Technology Co. Ltd., China). Contrast-to-noise ratio (CNR) (21), which refers to the relative difference of signal intensity between two kinds of tissues, was adopted to evaluate the quality of the CT image for rabbit tongue tissues and human TSCC samples as shown in **Supplement Figure 1**.

### H&E Staining and Pan-CK IHC Staining

All rabbit tongue tissues and human TSCC samples were embedded in paraffin using the standard method after I_2_-IK-enhanced micro-CT imaging. Then, a series of 4-µm sections were prepared according to the CT image data. For H&E staining, all sections were stained using an automatic pathological section staining machine. For pan-CK IHC staining, paraffin sections were deparaffinized and dehydrated using a series of graded ethanol solutions. For antigen retrieval, the sections were heated in a microwave oven with a 10-mM citrate buffer solution (pH = 6) for 10 min. Endogenous peroxidase activity was quenched by incubating the sections in 0.3% H_2_O_2_ for 5 min. After blocking with 3% bovine serum albumin for 1 h at room temperature, the sections were incubated with anti-pan-CK (1:400, Cat. #ab80826, Abcam) primary antibodies overnight at 4°C and gently washed three times with PBS. Sample sections were then incubated with goat-antimouse secondary antibody (1:10,000, Cat. #ab205719, Abcam) for 2 h at room temperature. The signal was developed using the HRP substrate 3, 3’-diaminobenzidine (DAB). Nuclear counterstaining was performed using hematoxylin.

### Quality Assessment of H&E Sections

The quality of H&E sections was scored by two experienced pathologists in a blinded manner according to the Chinese standards for the quality control of pathological sections. The scoring criteria are shown in **Supplement Table 1**.

### Tissue Volume/Area Measurement

Before micro-CT imaging, the volume of tissue blocks was measured by drainage. During micro-CT imaging, their volume was measured by 3-D reconstruction with the SeProcessPro Version.1 software. To evaluate the consistency of volume measurement between the drainage and 3-D reconstruction methods, 12 rabbit tongue tissues were measured using both methods. The results showed consistency between the two methods (*p* = 0.829) (**Supplement Figure 2**).

For determining tissue shrinkage during the H&E staining process, we calculated the tissue area change using Image J (1.52a, National Institutes of Health, USA). Before H&E staining, the maximum area of the tissues was calculated. Then, the maximum tissue sections were cut and used for the subsequent staining process. After H&E staining, we calculated the area of the pathological section.

### I_2_-IK Cells Staining

The human TSCC line SCC9 was purchased from Procell Life Science & Technology Co., Ltd. (Wuhan, China). Cells were cultured in DMEM/high-glucose medium and DMEM/low-glucose medium, respectively, supplemented with 10% (v/v) fetal bovine serum, 1% (v/v) penicillin, and 1% (v/v) streptomycin for 1 week. Then, 5×10^5^ SCC9_high glucose_ and SCC9_low glucose_ cells in 5 ml were seeded on 6-well plates and incubated overnight under a 5% CO_2_ atmosphere at 37°C. Next, the treated cells were washed twice with PBS and fixed in a formalin solution for 30 min. Then, the treated cells were incubated with various concentrations of I_2_-IK (0%, 2%, 4%, and 8%, w/v, the concentration of iodine) for 1 h. Finally, the treated cells were observed by optical microscopy. The staining intensity was calculated using Image J (1.52a, Wayne Rasband, National Institutes of Health, USA).

### Glycogen Content

Six tumor tissues, muscle tissues, and mucosal tissues from patients with TSCC were acquired to determine the glycogen content using the liver/muscle glycogen assay kit (Nanjing Jiancheng Bioengineering Institute, China).

### Statistical Analysis

Statistical analysis was performed using the SPSS statistical software (version 23.0, IBM, Chicago, Illinois, USA). Values are presented as mean ± standard derivation (SD). Multivariate repeated-measures ANOVA was used to compare the CNR and volume shrinkage of tissue blocks with different concentrations of I_2_-IK. One-way ANOVA was used to compare the volume shrinkage and quality of pathological sections with different concentrations of I_2_-IK. Student’s *t*-test was used to compare the glycogen content between tumor tissues, muscle, and mucosa. *P* < 0.05 was considered statistically significant.

## Results

### 3% I_2_-IK Staining for 48 h Was the Most Optimal Application Parameter for Micro-CT Imaging of Rabbit Tongue Tissues

#### Volume Change in Rabbit Tongue Tissues During the Staining Process

**Figure 1:**
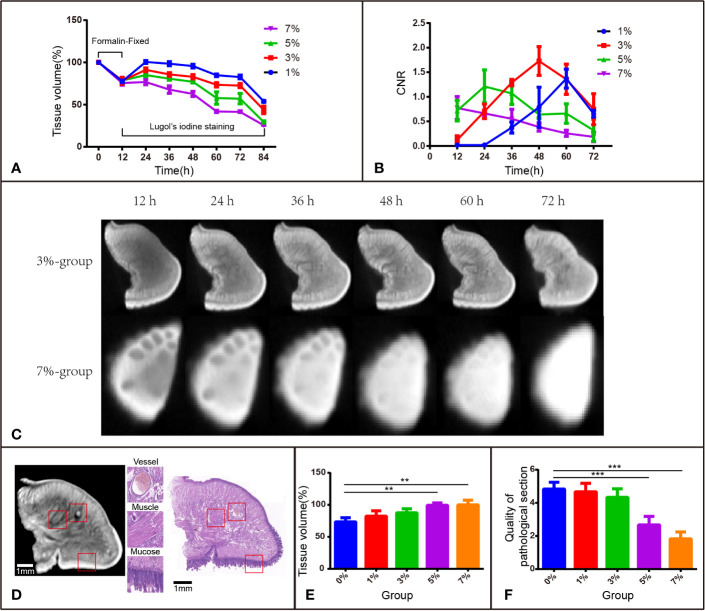
I_2_-IK-enhanced micro-CT image for rabbit tongue tissues. **(A)** Tissue shrinkage during formalin fixation and I_2_-IK staining (tissue volume normalized to 100%). **(B)** CNR value of micro-CT image at different times with different concentrations of I_2_-IK. **(C)** Micro-CT image data at different times with 3% and 7% I_2_-IK staining. **(D)** Micro-CT image and H&E image of the same sections (the structures in the red box indicate mucous, muscle fibers, and blood vessels). **(E)** Tissue shrinkage during H&E staining. **(F)** The quality of H&E images with different concentrations of I_2_-IK. (**P < 0.01, ***P < 0.001).

To investigate the volume change in rabbit tongue tissues during the entire staining procedure, 15 blocks of tongue tissues obtained from euthanized rabbits were divided into 5 groups according to I_2_-IK concentration (1%, 3%, 5%, 7%, and the formalin solution as the control). The mean volume of the fresh tissue blocks was 0.3867 cm^3^ ± 0.0284 cm^3^. Before I_2_-IK staining, all 15 blocks of tissues were fixed with formalin solution for 12 h, and the mean volume shrinkage was 22.3% ± 2.6% (**Figure 1A**). Then, during the 72 h of I_2_-IK staining, the tissue blocks in the 4 experimental groups were scanned every 12 h by micro-CT (**Figure 1C**) and the tissue volume change was determined. At sequential time points, the mean volume changes in the 1% group were 0.6% ± 1.7%, -1.5% ± 2.5%, -4.4% ± 2.2%, -15.2% ± 1.6%, -17.2% ± 2.2%, and -46.1% ± 0.9%, respectively; the mean volume changes in the 3% group were -8.8% ± 2.3%, -14.2% ± 2.2%, -17% ± 2.6%, -26.5% ± 2.6%, -27.4% ± 2.3%, and -56.1% ± 4.4%, respectively; the mean volume changes in the 5% group were -14.9% ± 1.9%, -19.4% ± 1.7%, -22.9% ± 1.3%, -42.2% ± 7.2%, -42.9% ± 6.2%, and -70.5% ± 1.3%, respectively; and the mean volume changes in the 7% group were -23.2% ± 3.2%, -32.2% ± 4.5%, -37.3% ± 3.0%, -58.1% ± 1.2%, -58.5% ± 0.7%, and -74.4% ± 0.2%, respectively (**Figure 1A**). Notably, the tissue blocks expanded in all groups in the first 12 h. In the following 60 h, the tissues began to shrink and showed a negative correlation between I_2_-IK concentrations and the shrinkage degree. Overall, low concentrations of I_2_-IK could reduce the shrinkage of tissue blocks and avoid the adverse effects of tissue shrinkage.

#### Micro-CT Image Quality During I_2_-IK Staining

I_2_-IK concentration and staining time could affect micro-CT imaging quality; thus, it is very important to ensure that the two application parameters are optimal. CNR was used as the standard to evaluate image quality (**Figures 1B, C**). During the staining period, there was a better-to-worse tendency of micro-CT imaging quality based on the CNR. The highest CNR was presented at 60 h for the 1% group (1.371 ± 0.192), at 48 h for the 3% group (1.729 ± 0.292), at 24 h for the 5% group (1.213 ± 0.337), and at 12 h for the 7% group (0.772 ± 0.233). Obviously, a shorter staining time was used to obtain good imaging quality for a higher I_2_-IK concentration. However, it is also easy to overstain with a high concentration of I_2_-IK. In summary, the CNR results showed that the highest imaging quality was obtained with 3% I_2_-IK staining for 48 h.

#### Pathological Sections and the H&E Staining Process

All tissue blocks in the 4 experimental groups were subjected to the standard H&E staining protocol and were compared with the control group (formalin). The H&E imaging quality and the area alteration of tissue sections (4 μm) were analyzed to evaluate the influence of I_2_-IK staining in subsequent pathological staining (**Figures 1E, F**). After scoring by two pathologists in a blinded manner, the quality of H&E sections was found to worsen after 5% and 7% I_2_-IK staining for 72 h (*P* < 0.001), whereas 1% and 3% I_2_-IK staining for 72 h had little influence compared with that in the control group (*P* > 0.05) (**Figure 1F**). Moreover, comparing of the micro-CT and H&E staining images suggested that 3% I_2_-IK-enhanced micro-CT imaging could clearly distinguish the microscopic structure including the mucous, muscle fibers, and blood vessels (**Figure 1D**). The area alteration of tissue sections was evaluated by calculating the area of the maximum section of tissue blocks before and after H&E staining. Unlike tissue shrinkage during I_2_-IK staining, a higher I_2_-IK concentration caused less alteration of the tissue section area as shown in **Figure 1E**.

After comprehensive consideration of the above-mentioned evaluation indicators, we could take a preliminary view that 3% I_2_-IK for 48 h was the optimal staining parameter for micro-CT imaging of rabbit tongue tissues.

### Staining With 3% I_2_-IK for 12 h Was the Best Parameter for Micro-CT Imaging of TSCC Samples

#### Volume Change of TSCC Samples During I_2_-IK Staining

**Figure 2:**
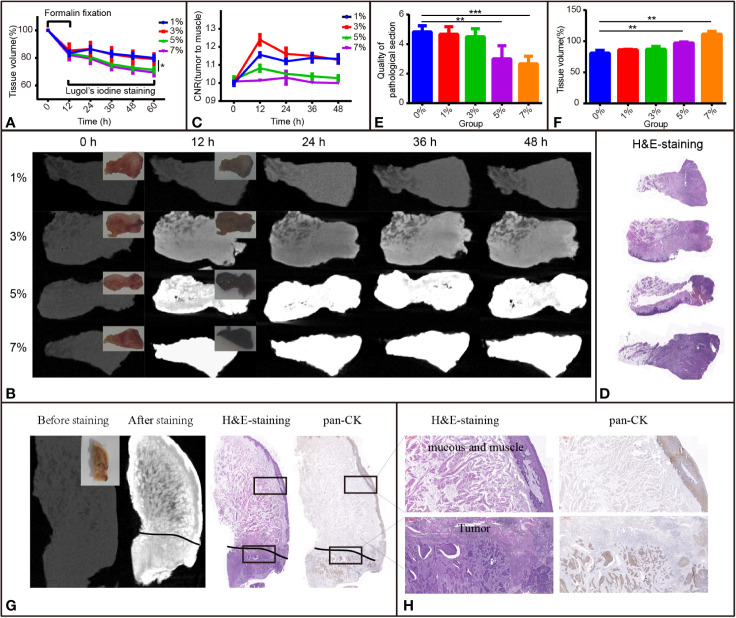
I_2_-IK-enhanced micro-CT images for TSCC samples. **(A)** Tissue shrinkage during formalin fixation and I_2_-IK staining time (tissue volume normalized to 100%). **(B)** Micro-CT image data at different times with different concentrations of I_2_-IK (insets indicate camera images of TSCC tumor tissue blocks). **(C)** CNR value of micro-CT images at different times with different concentrations of I_2_-IK. **(D)** H&E section of TSCC samples. **(E, F)** Tissue shrinkage and quality of pathological sections with different concentrations of I_2_-IK. **(G)** Micro-CT image before and after 3% I_2_-IK staining for 12 h and the H&E section, pan-CK section at the same level (black line indicates the tumor boundary). **(H)** Details of the H&E section and pan-CK section in the black boxes. (the above image shows mucous and muscle and the image below shows tumor tissues). **P < 0.01, ***P < 0.001.

To observe the influence of I_2_-IK concentrations and staining time on the volume change of TSCC samples, 12 samples were acquired and fixed in formalin solution for 12 h. The mean volumes of fresh samples were 0.8966 ± 0.0628 cm^3^, and the tissue after formalin fixation shrinkage was 16.42% ± 2.93% (**Figure 2A**). Then, the samples were stained with various concentrations of I_2_-IK (1%, 3%, 5%, and 7%) for 48 h. During the staining period, the samples were scanned by micro-CT every 12 h, and the volumes were determined by 3-D reconstruction. In detail, the mean volume changes in the 1% group were -13.7% ± 2.0%, -16.9% ± 1.8%, -18.4% ± 1.8%, and -20.1% ± 1.8%, respectively; the mean volume changes in the 3% group were -13.5% ± 4.8%, -17.4% ± 4.8%, -19.4% ± 4.4%, and -20.8% ± 3.9%, respectively; the mean volume changes in the 5% group were -19.3% ± 2.0%, -24.5% ± 1.6%, -27.1% ± 1.9%, and -28.6% ± 1.7%, respectively; and the mean volume changes in the 7% group were -20.5% ± 3.5%, -26.3% ± 2.2%, -28.6% ± 2.4%, and -30.5% ± 2.4%, respectively (**Figure 2A**). Similar to the rabbit tongue tissues, the volume of TSCC samples was also increased in the first 12 h and decreased at the latter time points. Furthermore, with the increase in I_2_-IK concentrations, the volumes of the tumor tissue blocks decreased more rapidly, suggesting that selecting low concentrations of I_2_-IK may reduce its adverse effects.

#### Micro-CT Image Quality During I_2_-IK Staining

To observe the effect of I_2_-IK concentration and staining time on the micro-CT imaging of TSCC samples, samples were scanned by micro-CT, and CNR values were calculated during I_2_-IK staining (**Figure 2B**). As shown in **Figure 2C**, the CNR value was first increased and then decreased at later times. Overall, the highest CNR was presented at 12 h for the 1% group (1.16 ± 0.01), at 12 h for the 3% group (1.24 ± 0.03), at 12 h for the 5% group (1.06 ± 0.02), and at 24 h for the 7% group (1.02 ± 0.04). Among these, 3% I_2_-IK staining for 12 h was the best condition to produce an optimal micro-CT image for TSCC samples.

#### Pathological Sections and H&E Staining

After I_2_-IK staining and micro-CT imaging, TSCC samples were further sectioned and subjected to H&E staining (**Figure 2D**). The quality of pathological images and area alteration of tissue sections was evaluated. As shown in **Figure 2E**, 5% and 7% I_2_-IK could decrease the pathological image quality, whereas 1% and 3% I_2_-IK had little influence compared to the control group. Moreover, an increase in I_2_-IK concentration showed increasing tissue section shrinkage as shown in **Figure 2F**. Furthermore, by comparing the micro-CT images and pathological images, we found that micro-CT imaging could clearly distinguish the boundary between tumor tissues and muscle tissues with 3% I_2_-IK staining for 12 h.

Overall, staining with 3% I_2_-IK for 12 h may be the optimal staining parameter for micro-CT imaging of TSCC samples.

### Micro-CT Imaging Could Clearly Distinguish Tumor Tissues From Normal Tissues After 3% I_2_-IK Staining for 12 h

After confirming the best parameters for micro-CT imaging of TSCC samples, 6 other TSCC samples were acquired to further demonstrate the feasibility of I_2_-IK-enhanced micro-CT in order to evaluate the status of the surgical margin in TSCC. These tissue blocks were fixed with formalin for 12 h, followed by staining with 3% I_2_-IK for 12 h. The tumor tissue blocks were scanned by micro-CT imaging before and after I_2_-IK staining (**Figure 2G**). Next, all tissue blocks were subjected to H&E and pan-CK IHC staining (**Figure 2G**). Upon comparing the micro-CT image, I_2_-IK-enhanced micro-CT image, H&E staining, and pan-CK staining, micro-CT could clearly distinguish the tumor tissues from normal tissues after staining with 3% I_2_-IK for 12 h (**Figures 2G, H**). Further, it had little influence on the subsequent pathological examination, including H&E and IHC staining.

### 3-D Comparison Between Micro-CT and H&E Staining Images

**Figure 3:**
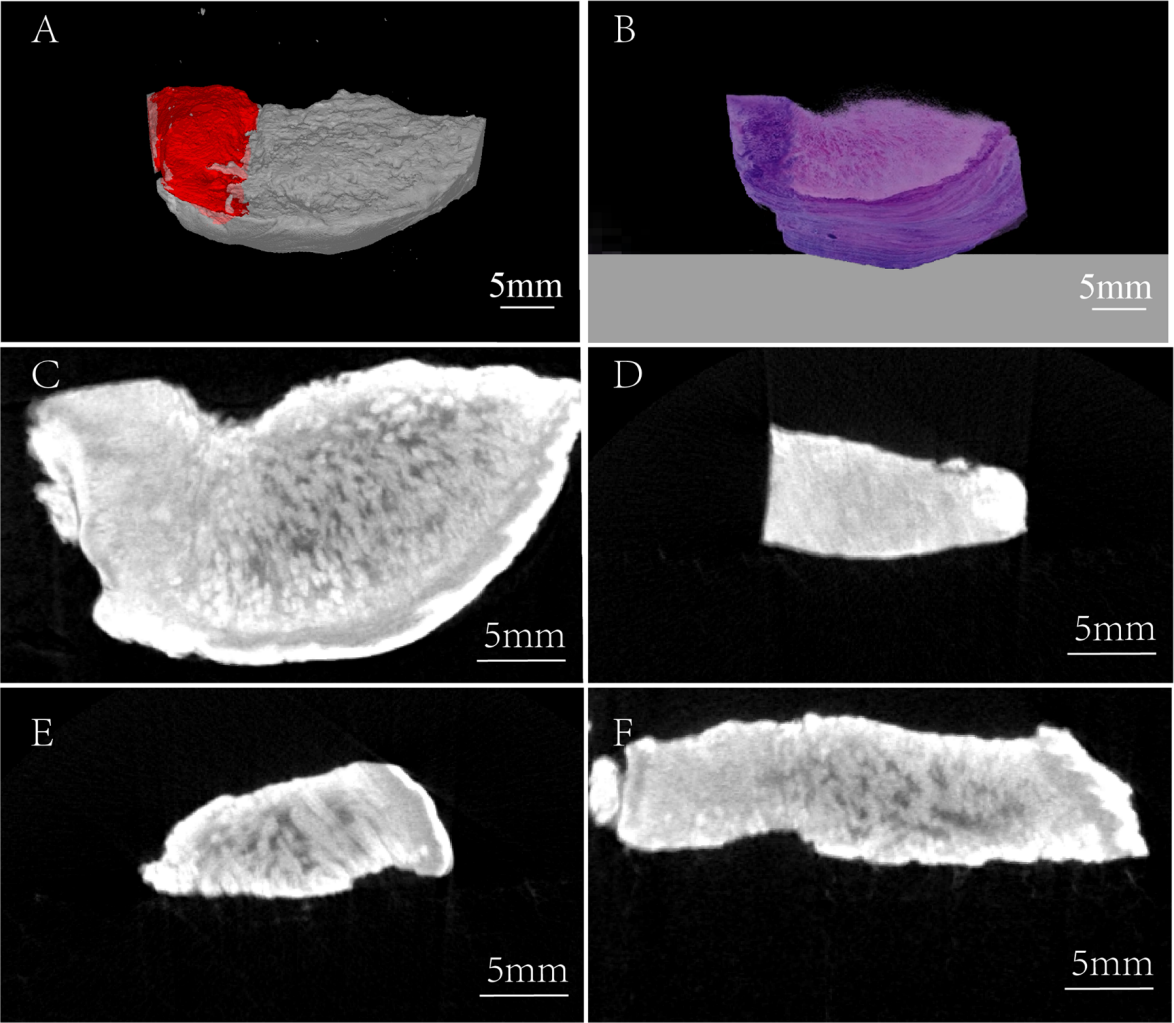
Three-dimensional comparison between the micro-CT image and H&E staining image. **(A)** Micro-CT 3-D reconstruction image, red region: tumor tissue. **(B)** 3-D H&E staining image. **(C)** Horizontal view of TSCC sample. **(D)** Frontal view of TSCC sample (the region of tumor tissue). **(E)** Frontal view of TSCC sample (the region of normal tissue). **(F)** Axial view of TSCC sample.

For further overall comparison between I_2_-IK-enhanced micro-CT imaging and subsequent pathological examination, the micro-CT 3-D regions of tumors were delineated by threshold segmentation using the SeProcessPro Version.1 software (**Figure 3A**), and semi-continuous paraffin sections were obtained at intervals of 50 µm and H&E staining was performed (**Figure 3B**). The results further verified that the I_2_-IK enhanced micro-CT image could clearly distinguish tumor tissues from normal tissues in a 3-D manner (**Figures 3C–F**).

### The Affinity of I_2_-IK With Different Tissues May Be Related to Glucose

**Figure 4:**
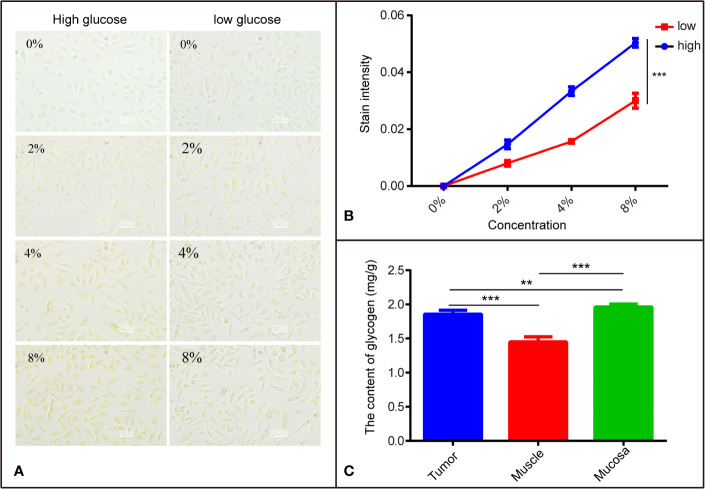
The different affinity of I_2_-IK with different tissues is related to glycogen. **(A)** The stained image of SCC9 cells with different concentrations of I_2_-IK. **(B)** Quantitative result of cells stained with I_2_-IK. **(C)** The content of glycogen in different tissues including tumor tissues, muscle, and mucosa. **P < 0.01, ***P < 0.001.

Previous studies (17) indicate that I_2_-IK has different affinities to different tissues, which may be related to glycogen. To verify the correctness of this hypothesis, SCC9 TSCC cells were cultured in DMEM-high and DMEM-low glucose medium, respectively. Then SCC9_high glucose_ and SCC9_low glucose_ were stained with different concentrations of I_2_-IK and observed by microscopy. The results show that I_2_-IK has more affinity with SCC9_high glucose_ compared to that with SCC9_low glucose_ (*p* < 0.001) (**Figures 4A, B**). Moreover, tumor tissues, muscle, and mucosa from a patient with OSCC were acquired to detect the glycogen content. The results show different contents of glycogen in tumor tissues, muscles, and mucosa (**Figure 4C**). This suggests that the mechanism underlying I_2_-IK-enhanced micro-CT is the different content of glucose in different tissues, resulting in different accumulation of I_2_-IK according to the tissue glucose content.

## Discussion

With spatial resolution reaching micron levels, micro-CT has been widely applied to various fields, including material science (22), archaeology (23), biomedicine (24–26), and so on. However, limited by density resolution, micro-CT is mainly used for hard tissue imaging, such as bone structure analysis in biomedicine. Notably, with the introduction of contrast agents such as I_2_-IK, the application scope of micro-CT has been further expanded to soft tissue imaging. Thompson et al. utilize I_2_-IK to stain the rat sciatic and pig vagus nerves for 24 and 120 h and show that the anatomy of peripheral nerves was clearly depicted by micro-CT imaging compared to the pathological sections (27). Jeffery et al. also illustrate micro-CT images in which contrast between muscle and connective tissues was achieved by means of staining with I_2_-IK (28). However, with the variety of stains gradually increasing, selection of proper stains is also vital for micro-CT imaging of TSCC samples. I_2_-IK, phosphomolybdic acid (PMA), phosphotungstic acid (PTA), and osmium tetroxide are the commonly used contrast agents at present. Among these, I_2_-IK is the most widely used for its advantages of low cost, convenient use, nontoxicity, and so on (24, 25). Compared to I_2_-IK, PMA- and PTA-enhanced micro-CT imaging yield similar imaging quality. However, application of PMA and PTA is limited by inevitable shortcomings, including irreversible tissue destruction, poor ability of tissue penetration, and exorbitant price. With osmium tetroxide, the image quality was relatively poorer and was mainly applicable to specimens embedded in resin blocks. Therefore, I_2_-IK has been adopted to enhance the contrast between OSCC tumors and normal tissues in this study. Compared to simple micro-CT imaging without staining, the fine anatomy in OSCC samples includes tumor, muscle, and mucosa tissues, which may be easier to distinguish after I_2_-IK staining.

Although I_2_-IK is an ideal contrast agent for micro-CT imaging of soft tissues, including tumor samples, overstaining or understaining may considerably affect the final image quality. The key to solving this problem is to explore the optimal staining parameters. I_2_-IK concentration and staining time are the two key parameters for micro-CT imaging quality. According to previous studies (29), different tissues have different optimal parameters for micro-CT. The optimal concentration of I_2_-IK ranged from <1% to 40%, whereas the staining time ranged from 30 min to 100 days for different kinds of tissues (24, 29, 30). To sum up, staining with low concentrations of I_2_-IK needs more time and produces little contrast between different tissues, whereas staining with high concentrations of I_2_-IK may lead to overstaining, which may reduce the contrast. Further, staining with a high concentration of I_2_-IK may further influence the quality of the pathological section/staining (31). In this study, the time- and concentration-dependent effects of I_2_-IK on the quality of micro-CT image and pathological section/staining were analyzed. We found that 3% I_2_-IK staining for 12 h was optimal for micro-CT imaging of OSCC primary tumor tissues.

At present, complete surgical excision of tumor lesions remains the first choice for patients with TSCC. However, due to the strong invasive characteristics of TSCC and the complex anatomical structure in the oral region, it is extremely difficult to remove TSCC tumor cells completely. The concept of a safe margin has, thus, been introduced and is continuously being improved. Currently, a safe margin is indicated by a distance of at least 5 mm away from the tumor tissues under microscopy, to indicate normal tissues (32). Intraoperative frozen sections and general paraffin-embedded sections have been used to evaluate the status of the surgical margin. However, due to the limitation of tumor tissue sampling, conventional pathological methods often produce false negatives, which lead to TSCC recurrence. In this study, we prove that I_2_-IK-enhanced micro-CT imaging could provide a complete and detailed 3-D anatomical structure of TSCC *in vitro* samples. Thus, with the help of I_2_-IK-enhanced micro-CT, conventional pathological examination of the status of the surgical margin will be more accurate. It is, thus, meaningful for clinicians to decide further treatment options for patients with OSCC after surgery.

Detection of residual tumor tissues intraoperatively and their timely resection may reduce the regional recurrence of TSCC (6). At present, there are two persistent key problems that need to be solved to promote the application of I_2_-IK-enhanced micro-CT image intraoperatively. First, the current definition of a safe margin is based on the distance of the pathological section. Owing to inevitable tissue shrinkage during the I_2_-IK staining process and pathological section preparation, it is important to accurately convert the distance between the pathological image and micro-CT image. In this study, tissue shrinkage during the I_2_-IK staining process and pathological section preparation process was calculated roughly to draw the following conclusions. The higher the I_2_-IK concentration, the more the tissue shrinkage during I_2_-IK staining process, whereas less tissue shrinkage occurred during pathological sectioning. However, these results are not sufficient for accurate distance conversion between a pathological image and micro-CT image. Nowadays, with the continued development of artificial intelligence (AI) technology, the combination of deep machine learning and a biomedical image processing algorithm like texture matching and nonrigid registration algorithms may provide a perfect solution (33–35). Second, in this study, OSCC tumor tissue blocks needed to be stained for 12 h, which hampers the application of I_2_-IK-enhanced micro-CT imaging during operation. Thus, accelerating the process of I_2_-IK staining is an important issue to resolve. Temperature is also an important factor affecting molecular motion and molecular diffusion. An increase in the staining temperature may improve the penetration rate of I_2_-IK into OSCC tumor samples. However, the structure and composition of OSCC tissues may also change as the temperature rises. Therefore, the optimal staining temperature remains to be explored. In addition to temperature, ultrasound also improves the motion and diffusion of molecules. Khosrawipour et al. found that, after 300 s of high-intensity ultrasound (HIUS) treatment, tissue penetration of doxorubicin into the peritoneal tissue improved by 3.8-fold (36). Further studies are thus needed to promote the application of I_2_-IK-enhanced micro-CT imaging intraoperatively.

## Conclusion

I_2_-IK-enhanced micro-CT imaging could position the tumor accurately and illustrate the tumor margin in TSCC specimens. It may, thus, be a potential method to evaluate the surgical margin and assist in margin sampling for patients with TSCC during surgery.

## Data Availability Statement

The original contributions presented in the study are included in the article/**Supplementary Material**. Further inquiries can be directed to the corresponding authors.

## Ethics Statement

The studies involving human participants were reviewed and approved by the medical ethics committee of the Institute Affiliated Stomatology Hospital, the Nanjing University Medical School. The patients/participants provided their written informed consent to participate in this study. The animal study was reviewed and approved by the Care Committee of the Nanjing University.

## Author Contributions

C-WX, R-lG, and Y-XW are responsible for the study concept and study design. C-WX, R-lG, J-rP, S-qH, and Q-zZ performed the data acquisition. C-WX, R-lG, SC, and LZ performed the quality control of data and algorithms, data analysis and interpretation, and statistical analysis. C-WX and R-lG prepared the manuscript. Y-XW and Q-GH edited and revised the manuscript. All authors contributed to the article and approved the submitted version.

## Funding

This study was supported by grants from the State Commission of Science & Technology of China (2016YFC0104100), the Jiangsu Province Science & Technology Department (BE2018618), and the Nanjing Medical Science and Technique Development Foundation (QRX17174).

## Conflict of Interest

The authors declare that the research was conducted in the absence of any commercial or financial relationships that could be construed as a potential conflict of interest.

## Abbreviations

3D, Three-dimensional; AI, Artificial intelligence; CNR, Contrast to Noise Ratio; CT, Computed tomography; DAB, Diaminobenzidine; H&E, Hematoxylin and Eosin; HIUS, High-intensity ultrasound; I_2_-IK, Lugol’s iodine; ICG, Indocyanine green; IHC, Immunohistochemistry; OSCC, Oral squamous cell carcinomas; PMA, Phosphomolybdic acid; PSM, Positive surgical margin; PTA, Phosphotungstic acid; TSCC, Tongue squamous cell carcinomas.
