# Supplementary material for: Lugol’s Iodine-Enhanced Micro-CT: A Potential 3-D Imaging Method for Detecting Tongue Squamous Cell Carcinoma Specimens in Surgery
Source: Front Oncol. 2020 Oct 21;10:550171. doi: 10.3389/fonc.2020.550171 (PMC7609877; doi:10.3389/fonc.2020.550171)
Supplement: Supplementary file 1 [file DataSheet_1.pdf]

Supplementary Material

1. Supplementary figures

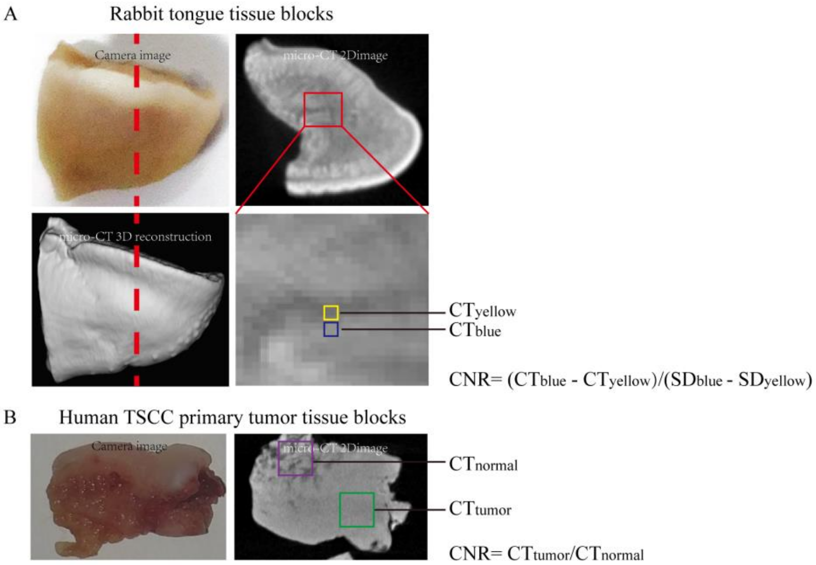

Supplementary Fig 1 Quality assessment of CT image. (A), CNR were adopted to evaluate the quality of micro-CT imaging for rabbit tongue tissues. (Yellow box shows the structure of muscle compartments and blue box shows the muscle structure) (B), CNR were adopted to evaluate the quality of micro-CT imaging for human TSCC samples. (Green box shows the structure of tumor tissues and purple box shows the structure of muscle tissues).

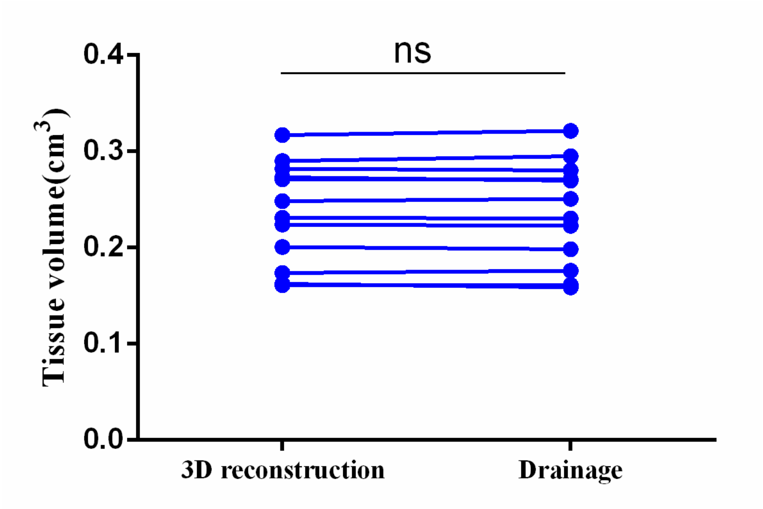

Supplementary Fig 2 The consistency of volume measured between the drainage and 3D reconstruction methods. Note: ns: no significance.

2. Supplementary table

Supplementary Table 1 Scoring criteria for H&E-stained sections

Score	Red and blue	Fold and	Contaminant	Diagnosis

	**contrast**	**Bubble**		
Poor quality (0-1)	Unclear	Present	Present	Difficult
Medium quality (2-3)	Clear	None	None	Easy
Good quality (4-5)	Very clear	None	None	Very easy

Notes: If the scores between two pathologists were different, the section was scored by a third pathologist.
